# Enhancing Targeted Colorectal Cancer Therapies with Natural Products: Mechanistic Pathways

**DOI:** 10.3390/biomedicines14071448

**Published:** 2026-06-26

**Authors:** Antonia Armega-Anghelescu, Daliborca Cristina Vlad, Calin Muntean, Corina Flangea, Flavia Zara, Mihai Mituletu, Tania Vlad, Victor Dumitrascu

**Affiliations:** 1 Doctoral School, Faculty of Medicine, “Victor Babes” University of Medicine and Pharmacy, 2nd Eftimie Murgu Square, 300041 Timisoara, Romania; antonia.armega@umft.ro (A.A.-A.); tania.vlad@umft.ro (T.V.); 2 Department of Microscopic Morphology, Faculty of Medicine, “Victor Babes” University of Medicine and Pharmacy, 2nd Eftimie Murgu Square, 300041 Timisoara, Romania; flavia.zara@umft.ro; 3 Department of Biochemistry and Pharmacology, Faculty of Medicine, “Victor Babes” University of Medicine and Pharmacy, 2nd Eftimie Murgu Square, 300041 Timisoara, Romania; vlad.daliborca@umft.ro (D.C.V.); dumitrascu.victor@umft.ro (V.D.); 4 “Pius Brinzeu” County Emergency Hospital, Liviu Rebreanu Blvd 156, 300723 Timisoara, Romania; 5 Department of Medical Informatics and Biostatistics, “Victor Babes” University of Medicine and Pharmacy, 300041 Timisoara, Romania; 6 ANAPATMOL Research Center, Department of Cell and Molecular Biology, Faculty of Medicine, “Victor Babes” University of Medicine and Pharmacy, 2nd Eftimie Murgu Square, 300041 Timisoara, Romania; mihai.mituletu@umft.ro

**Keywords:** metastatic colorectal cancer, targeted therapy, phytochemicals, gut microbiota, synergistic effect, cetuximab, bevacizumab, Fusobacterium nucleatum, chemoresistance, integrative oncology

## Abstract

**Background**: Colorectal cancer (CRC) remains a leading cause of mortality worldwide, with a significant proportion of patients presenting with metastatic disease (mCRC). While molecularly targeted therapies, including anti-EGFR and anti-VEGF agents, have improved survival outcomes, their efficacy is often limited by drug resistance, toxicity, and high costs. There is a growing need for sustainable strategies to enhance therapeutic efficacy. **Methods**: This review explores the emerging role of plant-derived compounds as synergistic adjuvants. Specifically, PubMed, Scopus, and Web of Science were searched for English-language articles published between January 2004 and June 2026, using combination of terms related to colorectal cancer, metastatic disease, anti-EGFR/anti-VEGF targeted therapy, phytochemicals/natural products, and gut microbiota; both primary studies and reviews were eligible. **Results**: Targeted therapies such as cetuximab and bevacizumab are the standard of care but face challenges related to *RAS*/*BRAF* mutations and primary tumour location. Clinical data demonstrate that while cetuximab improves overall survival in patients with *RAS* wild-type, left-sided tumours (median OS 31 vs. 26 months; HR 0.76, *p* = 0.012), progression-free survival remains comparable to that of bevacizumab. Concurrently, natural products like *Vitis vinifera*, *Dendrobium candidum*, and quercetin demonstrate significant preclinical potential in inhibiting angiogenesis, inducing apoptosis, and modulating the tumour microenvironment. The gut microbiome, particularly *Fusobacterium nucleatum* (whose reported prevalence varies widely across cohorts and reaches up to ~98% of CRC tissues only in selected series), has emerged as a key factor in chemoresistance. It should be emphasised that the great majority of the phytochemical-targeted therapy combinations discussed here are currently supported primarily by preclinical (in vitro and animal) studies rather than by clinical trials. **Conclusions**: Integrating evidence-based phytochemicals with conventional targeted therapies is a mechanistically compelling and potentially sustainable strategy that may enhance therapeutic efficacy, help overcome resistance, and mitigate adverse effects in mCRC management. However, because current support is largely preclinical, these combinations should be regarded as hypothesis-generating and require validation in prospective, biomarker-stratified clinical trials before clinical adoption.

## 1. Introduction

Colorectal cancer (CRC) represents the third-most frequently diagnosed malignancy (9.6% of all cancers) and the second leading cause of cancer-related mortality worldwide (9.3% of all cancer deaths), with close to 20 million new cancer cases and 9.7 million cancer deaths reported worldwide in 2022 [1]. It is estimated that by 2030, approximately 2.5 million new cases will be diagnosed [2]. In general, the 5-year survival rate is estimated to be 60% [3]. While the median age at diagnosis is 67 years, there is a concerning trend of a 2% annual increase in the incidence among patients younger than 50 [4]. At initial diagnosis, 15–30% of patients present with metastatic disease (mCRC), and a further 20–50% of those with localised cancer will subsequently develop metastases. The liver is the most common site of metastatic spread, followed by the lungs and peritoneum [5].

The five-year survival rate for patients with mCRC who do not receive treatment is as low as 3%, with a median life expectancy of only nine months [6]. The current standard of care for mCRC involves a combination of cytotoxic chemotherapy—typically fluoropyrimidine-based regimens like FOLFIRI (fluorouracil, leucovorin, irinotecan) or FOLFOX (fluorouracil, leucovorin, oxaliplatin)—with molecularly targeted biological agents [7,8].

A defining feature of CRC is its intimate and dynamic association with the gut microbiota, a critical component of the tumour microenvironment (TME). Accumulating evidence indicates that the gut microbiota, particularly species like *Fusobacterium nucleatum*, plays a pivotal role in CRC development, progression, and modulation of therapeutic responses [9,10]. *F. nucleatum* is frequently enriched within CRC tumours, with reported detection rates that vary widely across cohorts (commonly ~20–75%, and up to ~98% only in selected series), and has been shown to promote chemoresistance to standard agents like 5-fluorouracil (5-FU) and oxaliplatin by modulating autophagy [11,12]. It should be noted, however, that these associations are largely correlative and that reported prevalence depends strongly on the detection method, sampling, and cohort; a causal contribution of *F. nucleatum* to chemoresistance, although mechanistically plausible, therefore remains incompletely established and is addressed further in Section 3.4.

The selection of targeted therapy is guided by the molecular profile of the tumour. Key biomarkers, including mutations in *KRAS*, *NRAS*, and *BRAF* genes, as well as HER2 amplification and microsatellite instability (MSI) status, are mandatory for guiding first-line treatment decisions [5,13,14,15,16]. For patients with *RAS* wild-type (WT) tumours, monoclonal antibodies targeting the epidermal growth factor receptor (EGFR), such as cetuximab and panitumumab, are a cornerstone of therapy [17,18,19]. For others, agents targeting the vascular endothelial growth factor (VEGF) pathway, like bevacizumab, are employed to inhibit angiogenesis [20,21,22].

Despite these advances, challenges remain. The efficacy of anti-EGFR therapies is largely confined to patients with *RAS* WT, left-sided primary tumours, as demonstrated in landmark clinical trials like FIRE-3, which showed a significant overall survival benefit for cetuximab plus FOLFIRI over bevacizumab plus FOLFIRI in the per-protocol population (median OS 33 vs. 26 months; HR 0.75, *p* = 0.011) in this subgroup, despite no difference in progression-free survival [23]. Furthermore, drug resistance, significant toxicity, and high costs limit the long-term utility of these agents [24,25]. This has spurred research into novel strategies to enhance therapeutic efficacy and overcome resistance. Even though there are currently platforms for personalised medicine in CRC using digital and virtual twins, they require clinical validation [26]. One of the most promising avenues is the integration of natural products and phytochemicals, which can modulate multiple oncogenic pathways simultaneously, often with a more favourable safety profile [27,28,29,30]. Beyond their direct antitumor activity, many natural molecules display broad pleiotropic and health-promoting properties; notably, several can selectively amplify cytotoxicity in malignant cells while simultaneously protecting normal tissues from chemotherapy-associated organ injury, a dual effect that strengthens the rationale for their use as adjuncts to targeted therapy [31].

**Figure 1:**
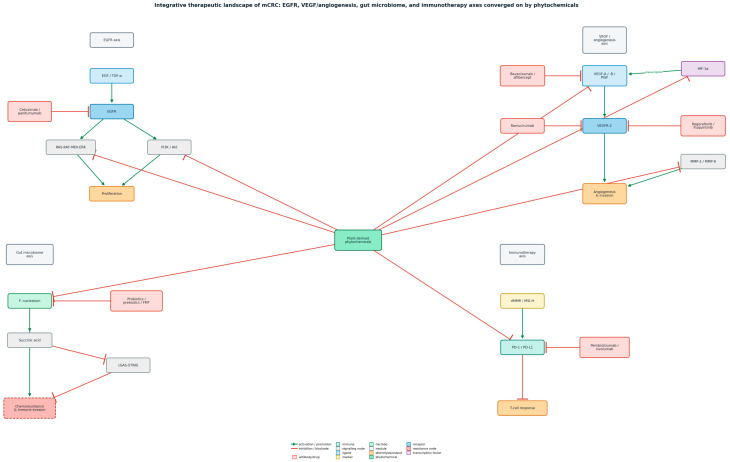
Integrative landscape of the four principal therapeutic axes in metastatic colorectal cancer (mCRC) and the molecular nodes at which plant-derived phytochemicals converge. EGFR axis: epidermal growth factor (EGF)/transforming growth factor-alpha (TGF-α) activate the epidermal growth factor receptor (EGFR), driving the RAS-RAF-MEK-ERK and PI3K/Akt cascades toward proliferation; the anti-EGFR antibodies cetuximab and panitumumab bind to and block EGFR. VEGF/angiogenesis axis: hypoxia-inducible factor-1alpha (HIF-1α) drives transcription of vascular endothelial growth factor (VEGF); bevacizumab neutralises the VEGF-A ligand and aflibercept acts as a decoy for VEGF-A/-B and placental growth factor (PlGF), whereas ramucirumab and the tyrosine kinase inhibitors regorafenib and fruquintinib block VEGF receptor-2 (VEGFR-2) signalling, thereby inhibiting angiogenesis and invasion; matrix metalloproteinases-2 and -9 (MMP-2/MMP-9) further promote angiogenesis and invasion. Microbiome axis: *Fusobacterium nucleatum* promotes chemoresistance and immune evasion, in part because its succinic acid suppresses the cGAS-STING innate immune pathway, and it is modulated by probiotics, prebiotics, and faecal microbiota transplantation (FMT). Immunotherapy axis: in mismatch-repair-deficient/microsatellite-instability-high (dMMR/MSI-H) tumours, programmed cell death protein-1/its ligand (PD-1/PD-L1) signalling suppresses the T-cell response, and the checkpoint inhibitors pembrolizumab and nivolumab block PD-1/PD-L1 to restore T-cell activity. Phytochemicals (grape seed proanthocyanidins, epigallocatechin gallate, curcumin, quercetin, resveratrol, Dendrobium/*Astragalus* polysaccharides, berberine) converge on HIF-1α/VEGF and MMP-2/9, PI3K/Akt and RAS-RAF, the microbiome, and the PD-1/PD-L1 immune checkpoint. Pointed arrows denote activation; blunt T-bars denote inhibition (The figure was generated with Inkscape, versions 0.92 and 1.0).

This review synthesises the current landscape of molecular targeted therapies for mCRC, evaluates the evidence for plant-derived compounds as synergistic adjuvants, and discusses the critical role of the gut microbiota in mediating treatment outcomes. Throughout, emphasis is placed on defined molecular targets—receptor tyrosine kinases, downstream RAS-RAF-MEK-ERK and PI3K/Akt effectors, mitochondrial apoptotic regulators (Bax/Bcl-2, caspases-3/9), matrix metalloproteinases, and the NF-κB transcriptional axis—and on the convergence of conventional and natural agents on these nodes, which provides the mechanistic basis for rational combination strategies (Figure 1). We propose that a multi-pronged approach, combining conventional targeted agents with evidence-based natural products, represents a sustainable and potent strategy for the future management of mCRC.

### Literature Search Strategy

This narrative review was based on a structured literature search of three electronic databases—PubMed, Scopus, and Web of Science—covering the period from January 2004 to June 2026 and restricted to peer-reviewed, English-language publications. Search strings combined controlled vocabulary and free-text terms for the disease (“colorectal cancer”, “metastatic colorectal cancer”), for conventional therapy (“cetuximab”, “panitumumab”, “bevacizumab”, “aflibercept”, “regorafenib”, “anti-EGFR”, “anti-VEGF”, “targeted therapy”), for natural agents (“phytochemical”, “natural product”, “plant extract”, and individual compound names such as curcumin, quercetin, resveratrol, and EGCG), and for the microbiota (“gut microbiome”, “*Fusobacterium nucleatum*”). Reference lists of retrieved articles and of recent reviews were screened manually for additional sources. Both primary experimental studies (in vitro, in vivo, and clinical) and review articles were eligible. As this is a mechanism-oriented narrative review rather than a systematic review, studies were selected on the basis of relevance to defined molecular targets, methodological quality, novelty, and translational value, with preference given to reports providing explicit molecular mechanisms or clinical trial data; non-English reports, conference abstracts without full text, and studies lacking a defined mechanism were generally excluded. No formal PRISMA-based quantitative screening or meta-analysis was performed, and this constraint is acknowledged in Section 4.3.

## 2. Molecular Targeted Therapies in mCRC: Current Landscape

The treatment paradigm for mCRC has shifted from a one-size-fits-all chemotherapy approach to a personalised strategy guided by the molecular characteristics of the tumour. The two most critical signalling pathways targeted in mCRC are the EGFR pathway, which drives cell proliferation, and the VEGF pathway, which fuels angiogenesis.

### 2.1. Anti-EGFR Therapy: Cetuximab and Panitumumab

Monoclonal antibodies targeting EGFR, such as cetuximab and panitumumab, function by blocking ligand binding to the receptor, thereby inhibiting the downstream RAS-RAF-MEK-ERK signalling cascade and preventing tumour cell growth and proliferation [32,33]. However, their efficacy is contingent on the tumour’s *RAS* gene status [34]. In general, cetuximab and panitumumab have similar efficacy, especially when used in combination with irinotecan [35]. Cetuximab is considered a first-line treatment for *KRAS*-WT EGFR mCRC [17] and has also been shown to inhibit VEGF [36,37]. Panitumumab is indicated for the treatment of *KRAS*-WT mCRC as monotherapy after chemotherapy failure or in combination with FOLFOX as a first-line treatment [38].

Biomarkers and Patient Selection. Mutations in *KRAS* and *NRAS* genes (found in approximately 50–55% of mCRC tumours) lead to constitutive activation of the downstream pathway, rendering anti-EGFR therapies ineffective [5,39,40]. Therefore, extended *RAS* mutation testing is mandatory before initiating treatment. The benefit of anti-EGFR therapy is almost exclusively seen in patients with *RAS* WT tumours [5,39].

Further stratification is provided by the primary tumour location. A substantial body of evidence, including post hoc analyses of the CRYSTAL and FIRE-3 trials, has demonstrated that the survival benefit from first-line anti-EGFR therapy is predominantly observed in patients with *RAS* WT tumours originating on the left side of the colon (distal colon and rectum). Patients with right-sided tumours derive little to no benefit and may even have worse outcomes with cetuximab compared to bevacizumab [23,41].

Clinical Efficacy. The addition of cetuximab to an FOLFIRI backbone has been extensively studied. The landmark CRYSTAL trial demonstrated that for patients with *KRAS* WT mCRC, adding cetuximab to FOLFIRI significantly improved progression-free survival (PFS) (HR 0.68; 95% CI, 0.50–0.94) and objective response rate (ORR) compared to FOLFIRI alone [39] (Table 1).

The FIRE-3 trial provided a head-to-head comparison of FOLFIRI plus either cetuximab or bevacizumab in patients with *RAS* WT mCRC. While there was no significant difference in the primary endpoint of PFS (10.0 vs. 10.3 months), the cetuximab arm showed a statistically significant and clinically meaningful improvement in the secondary endpoint of overall survival (OS) in the per-protocol analysis (median OS 33 vs. 26 months; HR 0.75; *p* = 0.011) for patients with left-sided tumours [23]. This suggests that anti-EGFR therapy provides a deeper and more durable response that translates into a survival advantage, particularly for patients who are candidates for curative-intent resection of metastases.

#### Acquired Resistance Mechanisms and ctDNA-Guided Rechallenge

**Figure 2:**
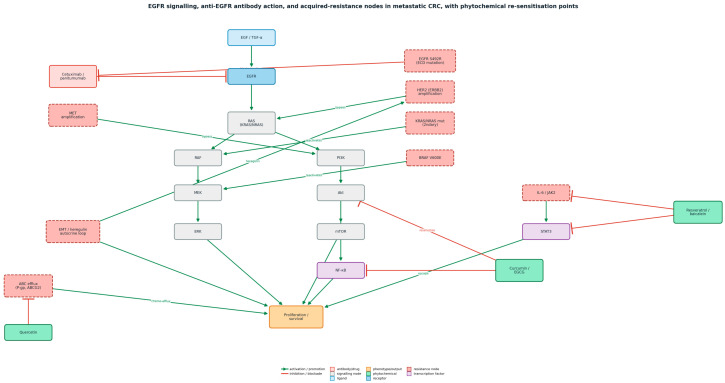
EGFR signalling in metastatic colorectal cancer (mCRC), the action points of anti-EGFR monoclonal antibodies, and the principal nodes of acquired resistance, with phytochemical re-sensitisation. EGF/TGF-α (transforming growth factor-alpha) engage EGFR (epidermal growth factor receptor), driving the RAS-RAF-MEK-ERK (mitogen-activated protein kinase) and PI3K-Akt-mTOR (phosphoinositide 3-kinase/mechanistic target of rapamycin) cascades, as well as NF-κB (nuclear factor kappa B), to promote proliferation and survival; clinical benefit of cetuximab/panitumumab requires *RAS* wild-type, left-sided tumours. Acquired resistance arises from secondary *KRAS*/*NRAS* mutations, *BRAF* V600E, HER2 (*ERBB2*) and *MET* amplification, the EGFR extracellular domain (ECD) mutation S492R that prevents antibody binding, EMT (epithelial–mesenchymal transition) with a heregulin (neuregulin-1) autocrine loop signalling through HER2/HER3, IL-6/JAK2/STAT3 (interleukin-6/Janus kinase 2/signal transducer and activator of transcription 3) escape, and ABC (ATP-binding cassette) efflux pumps (P-glycoprotein/ABCB1, ABCG2) that expel co-administered cytotoxic chemotherapy and thereby sustain tumour cell survival; the emergence and decay of these clones can be tracked by circulating tumour DNA (ctDNA)-guided rechallenge. Phytochemicals re-sensitise tumours through curcumin/EGCG (epigallocatechin gallate) suppression of NF-κB and PI3K/Akt, resveratrol/baicalein blunting of IL-6/STAT3, and quercetin inhibition of P-glycoprotein. Pointed arrows denote activation; blunt T-bars denote inhibition. (The figure was generated with Inkscape, versions 0.92 and 1.0).

Even in optimally selected *RAS* wild-type, left-sided tumours, virtually all patients who initially respond to cetuximab or panitumumab eventually develop acquired resistance. The dominant mechanisms are the emergence of secondary *RAS* and *BRAF* mutations, HER2 (*ERBB2*) amplification, *MET* amplification, and acquired mutations in the EGFR extracellular domain (most characteristically S492R) that sterically prevent antibody binding while preserving ligand-dependent signalling; reactivation of the RAS-RAF-MEK-ERK and PI3K/Akt cascades and an EMT/heregulin-driven autocrine loop also contribute [24,40,43,44]. Importantly, these resistant subclones are frequently polyclonal and, in the absence of continued anti-EGFR pressure, decay over time: circulating tumour DNA (ctDNA) studies show that mutant *RAS* clones expand under anti-EGFR therapy but progressively decline after its withdrawal, with an estimated half-life of approximately four months. This biology provides the rationale for ctDNA-guided anti-EGFR rechallenge, in which patients who progressed on first-line anti-EGFR therapy are re-treated only when a liquid biopsy confirms *RAS*/*BRAF*/EGFR wild-type status at the time of rechallenge (Figure 2). Prospective trials applying this molecular re-selection strategy (e.g., CHRONOS) have reported clinically meaningful response and disease control rates, establishing ctDNA-guided rechallenge as an actionable approach and a key research priority [24,40,45,46,47]. The pronounced benefit of anti-EGFR therapy in left-sided tumours and the lack of benefit (or potential harm) in right-sided tumours reflect these distinct molecular backgrounds: right-sided cancers are enriched for *BRAF* V600E, microsatellite instability, and a more mesenchymal, EGFR-independent biology, whereas left-sided cancers more often retain EGFR pathway dependency [48,49].

### 2.2. Anti-VEGF Therapy: Bevacizumab and Aflibercept

Angiogenesis, the formation of new blood vessels, is essential for tumour growth and metastasis. Bevacizumab, a monoclonal antibody, binds to and neutralises circulating VEGF-A, thereby preventing VEGF-A from engaging its receptors, VEGFR-1 and VEGFR-2, on endothelial cells rather than binding these receptors directly. Bevacizumab was the first anti-angiogenic agent to demonstrate a survival benefit in mCRC [50,51]. Its mechanism of action is independent of *RAS* or *BRAF* mutation status, making it a viable option for a broader patient population [52,53].

Clinical Efficacy. When added to standard first-line chemotherapy regimens (e.g., FOLFOX or FOLFIRI), bevacizumab consistently improves PFS by several months [54,55]. The pivotal AVF2107g trial showed that adding bevacizumab to IFL (irinotecan, fluorouracil, leucovorin) chemotherapy increased median OS from 15.6 to 20.3 months (HR 0.66, *p* < 0.001) [56]. While the OS benefit has been less pronounced with modern chemotherapy backbones, the improvement in PFS and response rates remains consistent, making it a standard first-line option, especially for patients with *RAS*-mutant tumours or right-sided primary tumours for whom anti-EGFR agents are ineffective.

Other anti-VEGF agents include aflibercept, a recombinant fusion protein that acts as a decoy receptor for VEGF-A, VEGF-B, and placental growth factor (PlGF), and regorafenib, a multi-kinase inhibitor. These are typically used in second-line or later settings after progression on first-line therapy [38,57,58,59]. Two additional agents have broadened the anti-angiogenic armamentarium. Ramucirumab, a fully human monoclonal antibody directed against the extracellular domain of VEGFR-2, prolongs overall survival when added to FOLFIRI after first-line progression (RAISE trial). Fruquintinib, a highly selective oral inhibitor of VEGFR-1/2/3, improved overall survival in heavily pretreated, chemorefractory mCRC irrespective of *RAS* status (FRESCO-2 trial) and received regulatory approval in 2023 [48,49,50]. A limitation common to all anti-angiogenic agents is adaptive (evasive) angiogenic escape: sustained VEGF/VEGFR blockade selects for compensatory up-regulation of alternative pro-angiogenic ligands—PlGF, fibroblast growth factors (FGF), and angiopoietin-2—and of HIF-1α-driven hypoxic signalling, together with increased pericyte coverage and vessel co-option, which collectively restore tumour neovascularisation and underlie acquired resistance to bevacizumab and multikinase inhibitors [24,58,59].

### 2.3. Mutational Landscape, Cytological Hallmarks, and Immunotherapy in Metastatic CRC

Beyond the well-defined RAS axis, the metastatic clone of CRC is shaped by a recurrent and partly mCRC-specific mutational architecture that conditions both prognosis and therapeutic response and is not interchangeable with the molecular profile of other carcinomas. *APC* loss-of-function (~75–80% of cases) initiates Wnt/β-catenin hyperactivation and is enriched at the invasive front; *TP53* mutations (50–60%) are strongly associated with the adenoma-to-carcinoma transition and with hepatic metastatic spread; *SMAD4* inactivation (10–20%) confers TGF-β pathway dysregulation and is an independent predictor of distant metastasis and of poor response to anti-EGFR antibodies. *KRAS* mutations (~40–45%, with codon 12/13 hot-spots and the emerging *KRAS* G12C subset) and *NRAS* mutations (~5%) are exclusive predictors of anti-EGFR resistance; *BRAF* V600E (8–12%) defines a biologically distinct, predominantly right-sided, microsatellite instability-prone subgroup with a median OS of barely 12 months on chemotherapy alone, for which the encorafenib + cetuximab combination (BEACON CRC) is now standard. *PIK3CA* mutations (15–18%) frequently co-occur with *RAS* alterations and engage the PI3K/Akt/mTOR axis as a parallel survival pathway. HER2 amplification (3–5%) and *NTRK* or *ALK*/*ROS1* fusions, although rare, define actionable subsets responsive to trastuzumab + tucatinib (MOUNTAINEER), trastuzumab–deruxtecan, or larotrectinib/entrectinib, respectively. *POLE*/*POLD1* ultra-mutated tumours and deficient Mismatch Repair/Microsatellite instability-High (dMMR/MSI-H) tumours (4–5% of mCRC) display a hypermutated phenotype with high tumour mutational burden and neoantigen load. Together with consensus molecular subtypes (CMS1–CMS4), these features define mCRC as molecularly heterogeneous, with cytological hallmarks—E-cadherin loss, vimentin/ZEB1/SNAI1-driven EMT, expansion of CD44+/CD133+/LGR5+ cancer stem-cell pools, dense stromal desmoplasia, and a *F. nucleatum*-enriched microenvironment—that anchor the rationale for the combinations discussed in Section 4 below [5,13,14,15,16,34,39,40,60]. The relative frequencies of these alterations are summarised in Table 2. The *KRAS* G12C variant (~3–4% of mCRC) is now specifically druggable: the covalent inhibitors sotorasib and adagrasib, which are largely ineffective as monotherapy in CRC because of rapid feedback EGFR reactivation, produce meaningful responses when combined with an anti-EGFR antibody—sotorasib plus panitumumab (CodeBreaK 300) and adagrasib plus cetuximab (KRYSTAL-1)—and this combination has now entered clinical practice [18]. More broadly, the mutational architecture of mCRC is not static but evolves under therapeutic pressure, with selection of resistant subclones. This molecular evolution can increasingly be tracked non-invasively by serial circulating tumour DNA (ctDNA) monitoring, enabling real-time detection of emerging *RAS*/*BRAF*/EGFR alterations and adaptive treatment switching [24,40].

Immunotherapy occupies a central but compartmentalised place in this landscape. In dMMR/MSI-H mCRC, immune checkpoint inhibitors targeting PD-1 (pembrolizumab, nivolumab) or PD-1 plus CTLA-4 (nivolumab + ipilimumab) are now the first-line standard of care: KEYNOTE-177 demonstrated that pembrolizumab doubled median PFS versus chemotherapy (16.5 vs. 8.2 months; HR 0.60, *p* = 0.0002), and CheckMate 8HW confirmed the superiority of nivolumab + ipilimumab over chemotherapy in the same population. Conversely, the ≈95% of mCRC patients with microsatellite-stable (MSS) disease show only minimal benefit from single-agent checkpoint blockade, reflecting low TMB, a “cold” immune contexture dominated by myeloid-derived suppressor cells, regulatory T cells, and tumour-associated macrophages, and active immune evasion mediated by *F. nucleatum*-derived succinic acid and by elevated TIGIT/CD155 and TIM-3/Galectin-9 axes. This explains why current trials (e.g., AtezoTRIBE, COMMIT, and MAYA) are testing PD-1/PD-L1 blockade combined with VEGF inhibition (bevacizumab) or with immunogenic chemotherapy/temozolomide priming, and why phytochemicals capable of remodelling the TME (curcumin, epigallocatechin-3-gallate (EGCG), baicalein, resveratrol, agrimonin, polysaccharides from Dendrobium and *Astragalus*, and berberine) are particularly attractive as immunomodulatory adjuvants in MSS mCRC, the very subgroup in which conventional immunotherapy currently fails [13,14,61,62,63,64,65].

## 3. Natural Products as Adjuvants and Synergistic Agents

The limitations of conventional targeted therapies—including toxicity, acquired resistance, and high cost—have fueled the search for complementary strategies. Plant-derived phytochemicals offer a compelling alternative, as they often possess multi-target capabilities, allowing them to modulate several oncogenic pathways simultaneously with potentially lower toxicity. This section explores the evidence for natural products as adjuvant agents in mCRC, structured by their primary mechanism of action.

### 3.1. Inhibition of Angiogenesis and VEGF Signalling

Several phytochemicals have demonstrated potent anti-angiogenic properties, mirroring the mechanism of agents like bevacizumab by targeting the VEGF signalling pathway.

*Vitis vinifera* (Grape Seed Extract): Proanthocyanidins from grape seed extract (GSE) have been shown to be potent inhibitors of angiogenesis [66]. Preclinical studies demonstrate that GSE can inhibit VEGF expression by reducing the protein expression of Hypoxia-Inducible Factor 1-alpha (HIF-1α), a key regulator of the cellular response to hypoxia [67,68]. By suppressing HIF-1α, GSE effectively downregulates VEGF production in cancer cells, thereby inhibiting endothelial cell proliferation, migration, and tube formation. Furthermore, GSE has been shown to reduce the expression of matrix metalloproteinases (MMPs), which are crucial for the degradation of the extracellular matrix during tumour invasion and metastasis [68,69]. Importantly, the molecular nodes engaged by GSE proanthocyanidins (HIF-1α→VEGF and MMP-2/-9) are precisely the targets of the molecularly targeted antiangiogenic therapies used in mCRC: bevacizumab and aflibercept neutralise VEGF-A (and, for aflibercept, additionally VEGF-B and PlGF), while regorafenib inhibits VEGFR-1/2/3 and platelet-derived growth factor receptor (PDGFR). GSE therefore does not act as an “alternative” antiangiogenic agent but as an upstream, transcriptional-level adjuvant that is mechanistically complementary to bevacizumab, aflibercept, and regorafenib, and preclinical evidence supports synergy with bevacizumab in HCT116 and SW480 xenografts (reduced microvessel density and tumour volume beyond either agent alone) [42,50,51,52,55,56,57,58,66,67,68,69].

*Coix lacryma-jobi* (Adlay): The seed extract of *Coix lacryma-jobi* (CLJ), known for its effects in CRC, has been traditionally used in Chinese medicine [70,71]. CLJ seeds contain a large number of compounds, such as proteins (e.g., coixin), flavonoids (especially quercetin), squalene and phytosterols with cytotoxic effects on cancer cells [70,72]. Modern research has shown that an extract of adlay seed (CLSE) can significantly inhibit key steps in angiogenesis [73,74]. In a seed mixture containing CLSE administered as a dietary supplement to mice, reduced activation of the VEGF-C/D-VEGFR-2/3 cascade was observed [73]. In previous studies using human umbilical vein endothelial cells, CLSE was found to inhibit the secretion of angiogenic factors and prevent the formation of capillary-like tubes, a hallmark of angiogenesis [74,75]. This effect suggests its potential to starve tumours of their required blood supply, similar to that of conventional anti-VEGF therapies [74].

Synergy with anti-angiogenic molecular targeted therapies. The phytochemicals discussed act on the same molecular axis exploited by the anti-VEGF/VEGFR drugs approved in mCRC: bevacizumab (anti-VEGF-A), aflibercept (anti-VEGF-A/-B/PlGF decoy), ramucirumab (anti-VEGFR-2), and the multikinase inhibitors regorafenib and fruquintinib (VEGFR-1/2/3, PDGFR, fibroblast growth factor receptor (FGFR), and TIE-2). Whereas the targeted agents neutralise circulating ligands or block receptor kinase activity, these phytochemicals act upstream by suppressing HIF-1α-driven transcription of VEGF, FGF-2, ANG-2, and PlGF and by downregulating MMP-2/-9. In preclinical mCRC models, this transcriptional-level inhibition prevents the compensatory ligand surge that drives acquired resistance to bevacizumab and regorafenib, providing a mechanistically rational basis for combining these phytochemicals as adjuvants to bevacizumab + FOLFOX/FOLFIRI in first-line mCRC and to aflibercept- or regorafenib-based regimens after progression [42,50,51,52,55,56,57,58,66,67,68,69]. It should be emphasised that the synergy described in this subsection derives exclusively from preclinical work. Effects demonstrated in endothelial or CRC cell cultures (e.g., suppression of capillary-tube formation and reduced VEGF secretion) are distinguished here from those confirmed in animal xenografts (e.g., reduced microvessel density and tumour volume), and no clinical trial has yet tested these phytochemical–bevacizumab combinations. Whether the active concentrations used in vitro are achievable in human plasma remains uncertain and is discussed in Section 4.1 and Section 4.3.

### 3.2. Induction of Apoptosis and Cell Cycle Arrest

Inducing apoptosis (programmed cell death) and arresting the cell cycle are cornerstone strategies in cancer therapy. Many phytochemicals excel in this area, often activating pathways that are dysregulated in cancer cells.

*Dendrobium candidum*: Polysaccharides from this orchid, a valued component of traditional medicine, have demonstrated significant anti-tumour activity [76,77]. *D. candidum* also contains a mixture of polyphenols, the most abundant being syringic acid, 4-hydroxybenzene acid and p-hydroxycinnamic acid, which contribute to its overall antitumour effect [77,78]. Another compound that may play a role in CRC treatment is erianin. Erianin has been shown to inhibit tumor growth in vivo by interfering with β-catenin [79,80]. Studies on CRC cell lines show that Dendrobium extracts can induce apoptosis by modulating the Bax/Bcl-2 ratio. An increase in the pro-apoptotic protein Bax and a decrease in the anti-apoptotic protein Bcl-2 lead to the activation of the mitochondrial apoptotic pathway, culminating in the activation of executioner caspases 3 and 9 [81].

Curcumin (from *Curcuma longa*): Curcumin is one of the most extensively studied phytochemicals. Its anticancer effects are mediated through the regulation of numerous signalling pathways. In cancer cells, curcumin induces apoptosis and inhibits proliferation by suppressing the NF-κB, PI3K/Akt, and MAPK signalling pathways [82,83,84,85,86]. Despite its potent preclinical activity, curcumin’s clinical utility has been hampered by its poor oral bioavailability [87]. A clinical study in CRC patients by Sharma et al. found that while oral doses up to 180 mg/day were safe, curcumin was barely detectable in the blood, though it was recovered in faeces, suggesting that it may exert its effects locally in the colon [88]. New formulations enhance the pharmacokinetics of curcumin, resulting in better absorption and distribution, as well as reduced degradation during metabolic transformations [87,89,90,91,92]. The local activity of curcumin in the colon when consumed as a spice is particularly relevant for preventing recurrence and modulating the gut microenvironment. To overcome the low systemic bioavailability that results from poor aqueous solubility, rapid metabolism, and fast elimination, a range of advanced delivery systems has been developed, including liposomes, polymeric and lipid nanoparticles, polymeric micelles, phytosomes, nanoemulsions, and cyclodextrin- or piperine-based co-formulations. In preclinical models, these can raise plasma and intratumoural curcumin concentrations several-fold and improve antitumour activity (discussed further in Section 4.1) [89,90,91,92,93,94,95]. Even so, such formulations remain largely preclinical, and the documented safety of high oral curcumin doses does not by itself guarantee that therapeutic systemic exposure is reproducibly achievable in patients.

Quercetin. This flavonoid, abundant in many fruits, vegetables, and honey, has shown promise in CRC treatment. It induces apoptosis by inhibiting the NF-κB pathway and generating reactive oxygen species (ROS) within cancer cells [96]. A meta-analysis of animal models has shown that quercetin reduces the number of aberrant crypt foci [97]. A synergistic effect has also been observed when quercetin is administered with chemotherapy, increasing the toxicity of doxorubicin in SW620/Ad300 colon cancer cells [98,99]. When used in combination with sulforaphane, quercetin enhances the action of oxaliplatin in the HCT116 cell line xenografted into BALB/c nude mice [100]. An additive effect was observed when 12 µM quercetin and 100 µM 5-FU were used on the p53-mutated HCT-15 cell line or when 580 µM quercetin was added to 5-FU in the HT-29 cell line [101]. In CRC cell lines such as CACO-2 and SW-620, quercetin has demonstrated IC50 values in the range of 20–35 μM. It also enhances the apoptotic effects of chemotherapeutic agents like 5-FU, particularly in MSI colorectal cancer cells, suggesting synergistic potential [102]. Importantly, these in vitro IC50 values (20–35 µM) and the synergy concentrations used above (up to ~580 µM) greatly exceed the low-micromolar (typically <2 µM) plasma levels attainable with oral quercetin in humans; such effects are therefore likely to be reproducible only with nano-enabled delivery or within the colonic lumen rather than at systemic sites. Quercetin is generally well tolerated, but high-dose or intravenous administration has been associated with pro-oxidant effects and, rarely, nephrotoxicity, which underscores the need for formal toxicity and safety evaluation (Section 4.1 and Section 4.3) [103,104].

*Paris polyphylla*. Saponins extracted from *Paris polyphylla* have been found to induce potent ROS-mediated apoptosis in CRC cells. The increase in intracellular ROS triggers oxidative stress, leading to mitochondrial dysfunction and the activation of caspase-3, a key executioner of apoptosis. In addition, they act synergistically with 5-FU and cisplatin against the HCT116 cell line [105]. Furthermore, fruits extracts exhibit an inhibitory effect on mitochondrial fusion induced by *Fusobacterium nucleatum*-derived extracellular vesicles. This effect was observed in the Caco-2 cell line [106], which is relevant because *F. nucleatum* is known to promote CRC progression and metastasis [107,108,109]. Additionally, polyphyllin I—a saponin found in *Paris polyphylla* [110]—induces apoptosis and G2 cell cycle arrest in a dose-dependent manner [111] while also exhibiting antiangiogenic effects [110].

*Cannabis sativa*. Cannabinoids, the active compounds in *Cannabis sativa*, have been shown to induce cell cycle arrest and apoptosis in CRC cells [61]. Cannabidiol (CBD), for example, activates AMP-activated protein kinase (AMPK), which in turn downregulates key survival pathways, including the phosphorylation of p38, ERK, and JNK, leading to reduced cell viability [112]. Furthermore, the anti-inflammatory effects of cannabidiol, such as inhibition of cyclooxygenase-2, and the reduction in nuclear factor-kB (NF-κB) activity induced by cannabigerol and tetrahydrocannabinol [113], may also contribute to the overall antitumor effect. Terpenes also contribute to this effect, especially β-caryophyllene, which has antiproliferative and proapoptotic effects on HCT116 CRC cells [114,115]. A synergistic effect was also observed when cisplatin and a dried flower extract—high in tetrahydrocannabinol (THC) (10%), β-myrcene, and α-pinene, and low in CBD (<1%)—were administered simultaneously to HT29 and HCT116 cells [116].

Synergy with anti-EGFR, anti-BRAF, anti-HER2 molecular targeted therapies. The apoptosis- and cell cycle-acting phytochemicals reviewed in Section 3.2 converge on signalling nodes that are also engaged by the molecularly targeted agents currently approved for mCRC: cetuximab and panitumumab (anti-EGFR mAbs), the encorafenib + cetuximab combination (*BRAF* V600E), and trastuzumab + tucatinib or trastuzumab–deruxtecan (HER2-amplified mCRC). Curcumin and EGCG suppress NF-κB and PI3K/Akt signalling downstream of EGFR and resensitise *KRAS*- or *BRAF*-mutant CRC cells to cetuximab and to encorafenib + cetuximab. Quercetin and sulforaphane potentiate cetuximab- and panitumumab-induced apoptosis by amplifying the Bax/Bcl-2 ratio and caspase-3/9 activation in HCT116, HT-29, and SW480 lines. Resveratrol and baicalein attenuate the IL-6/STAT3 escape pathway that drives secondary resistance to anti-EGFR therapy. Silibinin downregulates HER2 phosphorylation and synergises with trastuzumab in HER2-amplified CRC organoids. Importantly, these phytochemical-mediated effects on apoptosis and cell cycle arrest are not redundant with the cytostatic action of cetuximab/encorafenib but are complementary, reactivating the intrinsic apoptotic programme that targeted therapies engage only partially [39,70,71,72,82,83,84,85,86,96,97,98,99,100,101,102].

### 3.3. Suppression of Metastasis, Invasion, and Epithelial–Mesenchymal Transition (EMT)

**Figure 3:**
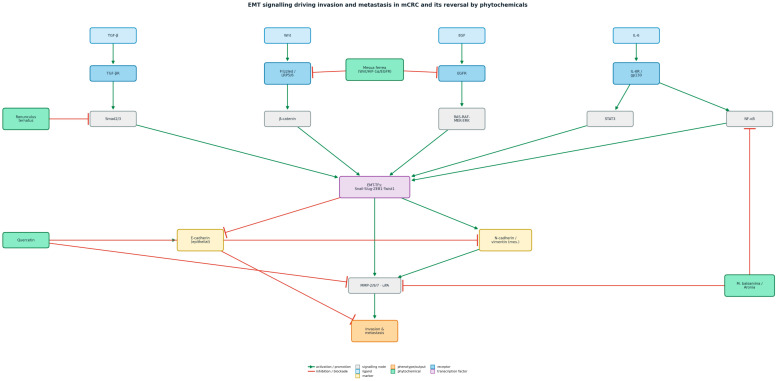
Epithelial–mesenchymal transition (EMT) signalling pathways driving invasion and metastasis in metastatic colorectal cancer (mCRC) and the phytochemicals that reverse them. Four extracellular inputs converge on the EMT programme: transforming growth factor-beta (TGF-β) acting through its receptor (TGF-βR) and Smad2/3; Wnt acting through Frizzled/LRP5/6 and β-catenin; epidermal growth factor (EGF) acting through the epidermal growth factor receptor (EGFR) via the RAS-RAF-MEK-ERK cascade; and interleukin-6 (IL-6) acting through IL-6R/gp130 to activate STAT3 (signal transducer and activator of transcription 3) and the inflammatory transcription factor NF-κB (nuclear factor kappa-B). These effectors activate the EMT transcription factors (EMT-TFs) Snail, Slug, ZEB1 and Twist1, which repress the epithelial marker E-cadherin while inducing the mesenchymal markers N-cadherin and vimentin and upregulate matrix metalloproteinases (MMP-2, MMP-9, MMP-7) and urokinase plasminogen activator (uPA) to promote invasion and metastasis; loss of E-cadherin further relieves the adhesion-mediated brake on these processes. Inhibitory (T-bar) actions show phytochemical reversal of EMT: quercetin restores E-cadherin and lowers N-cadherin/vimentin and MMP-2/9; *Mesua ferrea* targets Wnt/HIF-1α (hypoxia-inducible factor 1-alpha)/EGFR signalling; *Ranunculus ternatus* blocks TGF-β/Smad; and *Momordica balsamina* and Aronia suppress MMP-2/9 and NF-κB. (The figure was generated with Inkscape, versions 0.92 and 1.0).

Metastasis is the primary cause of mortality in CRC patients. It is a complex process involving cell invasion, migration, and the transition of epithelial cells to a mesenchymal phenotype (EMT), which grants them migratory and invasive properties [117,118,119]. Several phytochemicals have been found to interfere with these critical steps [75] (Figure 3).

*Mesua ferrea* (Ceylon Ironwood): Extracts from this plant have shown potent anti-metastatic activity. They inhibit cancer cell invasion and migration by modulating key signalling pathways implicated in metastasis, including WNT, HIF-1α, and EGFR, as demonstrated in HCT116 cell line [120,121]. The effect is attributed to terpenes extracted from the stem bark, the most abundant of which are α-amyrin and globulol [121]. When essential oils are used, the oleo-gum resin fraction—containing isoledene and α-elemene—downregulates survivin and X-linked inhibitor of apoptosis protein (xIAP) while caspase 3/7 and TRAIL-R2 are upregulated, activating ROS-mediated apoptosis [122,123]. By downregulating these pathways, *Mesua ferrea* can suppress the cellular machinery required for cells to detach from the primary tumour and invade surrounding tissues [120].

Quercetin and *Aronia melanocarpa* (Chokeberry): Quercetin demonstrates significant anti-metastatic effects by reducing the expression and activity of MMP-2 and MMP-9, enzymes that are essential for degrading the extracellular matrix [124,125]. Furthermore, it directly modulates EMT by increasing the expression of the epithelial marker E-cadherin while decreasing the mesenchymal marker N-cadherin, effectively reversing the metastatic phenotype [124]. The combination of 5-FU and Aronia berry extract not only reduces the required dosage of 5-FU but also decreases the viability, clonogenic potential, migration, and invasion of CRC cell lines (HCT116 and SW480). This effect is driven by the downregulation of Toll-like receptor 3 (TLR3) and NF-kB expression [126]. Extracts from *Aronia melanocarpa*, which are rich in polyphenols including quercetin, have also been shown to inhibit cancer cell motility [124,125].

*Ranunculus ternatus*: This plant regulates the TGF-β/Smad/EMT axis, a central pathway controlling EMT. By inhibiting this pathway, extracts from *Ranunculus ternatus* can prevent cancer cells from acquiring migratory capabilities. It also decreases the levels of the chemokine CCL5, which is involved in recruiting immune cells that can inadvertently support tumour progression [127]. The effect is likely due to the synergistic action of its constituents, including flavonoids, glycosides, amino acids, and sterols—specifically sterulin, linocaffeine, kayaflavone, and bilobetin [128].

*Momordica balsamina* (Balsam Apple): This plant exerts its anti-metastatic effects by downregulating the expression of MMP-2, MMP-9, and the transcription factor NF-κB. Since NF-κB is a master regulator of inflammation and cell survival, its inhibition has pleiotropic anti-cancer effects, including the suppression of invasion and metastasis [129]. The effect was evident in vitro, using the HT-29 cell line [129]. Furthermore, a synergistic effect with doxorubicin was demonstrated, mediated primarily by the triterpenes balsaminal and balsaminol G [130].

Synergy with molecular targeted therapies acting on invasion and metastasis. The anti-EMT, anti-MMP, and anti-Wnt phytochemicals act on molecular nodes that are exploited by current and emerging mCRC-targeted agents: bevacizumab and aflibercept (which restrict not only angiogenesis but also VEGF-driven invasion of liver sinusoids), regorafenib (which blocks PDGFRβ on stromal pericytes and TIE-2 on endothelial cells, both critical for the EMT–stromal interface), and the developmental Wnt/β-catenin and TGF-β inhibitors entering clinical trials in mCRC. By suppressing Snail/Slug/ZEB1, restoring E-cadherin, and downregulating MMP-2/-9, MMP-7, and uPA, these phytochemicals counteract precisely the EMT- and matrix-remodelling programmes that drive secondary resistance to bevacizumab and underlie hepatic metastatic colonisation. Combination with cetuximab in left-sided *RAS* WT mCRC is mechanistically rational because EGFR signalling itself activates Snail/ZEB1; phytochemical EMT suppression therefore extends rather than duplicates the anti-proliferative action of cetuximab [124,126,127,128,129,130]. The translational feasibility of these anti-EMT effects nonetheless remains uncertain: the supporting evidence is almost entirely from cell-line and xenograft models, the active concentrations frequently exceed clinically achievable systemic exposures, and the findings are not uniformly consistent—some reports describe context-dependent or even pro-migratory effects of individual polyphenols at particular doses or in specific genetic backgrounds. These conflicting data, together with the absence of clinical confirmation, should temper the interpretation of the anti-metastatic potential described above.

### 3.4. Modulation of Tumour Microenvironment and Gut Microbiota

The tumour microenvironment (TME), including its complex community of immune cells and microbes, is now recognised as a critical determinant of cancer progression and therapeutic response. The gut microbiota, in particular, has a profound impact on CRC. Dysbiosis, an imbalance in the gut microbial community, is a hallmark of CRC [131,132,133]. Several phytochemicals and traditional medicine formulations exert their anti-cancer effects by favourably modulating the TME and gut microbiota.

*Fusobacterium nucleatum* and Chemoresistance: As previously noted, *F. nucleatum* is highly prevalent in CRC tissues, with some studies reporting detection in up to 98.2% of cases, at levels 4- to 5-fold higher than in adjacent normal tissue [11]. Its presence is strongly associated with a worse prognosis, increased recurrence risk, and resistance to chemotherapy [62,134,135]. *F. nucleatum* promotes chemoresistance to agents like 5-FU and oxaliplatin by activating cytoprotective autophagy in cancer cells, thereby shielding them from drug-induced apoptosis [12,136]. Other studies suggest that *F. nucleatum* chemically modifies 5-FU by converting it to dihydrofluorouracil via the enzyme dihydropyrimidine dehydrogenase [137]. Furthermore, resistance to biological therapy with pembrolizumab and nivolumab–two programmed cell death protein-1 (PD-1) inhibitors—has been reported [63,64]. This has been attributed to the release of succinic acid into the cellular environment by *F. nucleatum*, which impairs the response to these drugs. Succinic acid downregulates the cGMP-AMP synthase–interferon β pathway, leading to decreased release of the chemokines CCL5 and CXCL10 [62,63]. This makes the targeted modulation of the gut microbiota a highly attractive therapeutic strategy. Two caveats temper these mechanistic data. First, much of the human evidence linking *F. nucleatum* to chemoresistance and poor prognosis is correlative, derived from association studies whose results vary with detection method, sampling, and cohort, so a causal role—although supported by mechanistic and animal work—is not firmly established. Second, the pathogenic signal appears to be clade-specific: recent genomic analyses indicate that the *F. nucleatum* animalis (Fna) C2 clade is selectively enriched in CRC and carries the relevant virulence and metabolic traits, whereas other clades are not, which may partly explain the wide variation in reported prevalence and effect size across studies [11,64].

Microbiome-directed interventions: Beyond phytochemicals, several strategies aim to therapeutically reshape the CRC-associated microbiota. Probiotics (e.g., selected *Lactobacillus* and *Bifidobacterium* strains) and prebiotics (fermentable fibres such as inulin and resistant starch) can promote short-chain-fatty-acid-producing taxa and may attenuate *F. nucleatum* colonisation and mucosal inflammation. Postbiotics—non-viable bacterial products and metabolites, most notably butyrate—exert direct anti-inflammatory and epithelial-protective effects and can enhance chemo- and immunotherapy responses in preclinical models. Faecal microbiota transplantation (FMT) is being explored to restore a favourable community structure and, in early phase studies in other tumour types, to overcome immunotherapy resistance. These approaches remain investigational in mCRC: clinical evidence is still limited, optimal formulations, dosing, and endpoints are undefined, and rigorous trials incorporating microbiome endpoints are required before integration into standard care [9,10,135,138,139,140].

Yanghe Decoction: This traditional Chinese medicine formulation has been shown to improve the gut microbial landscape in preclinical models of CRC. Treatment with Yanghe decoction leads to an increased abundance of beneficial *Bacillus* species. Concurrently, it potentiates systemic anti-tumour immunity by boosting the activity of Natural Killer (NK) cells and increasing levels of Interleukin-21 (IL-21), a cytokine crucial for coordinating anti-cancer immune responses [65,141]. Moreover, this mechanism contributes to the inhibition of pulmonary metastasis in CRC [141].

*Agrimonia pilosa* (Hairy Agrimony): This plant targets a key component of the immunosuppressive TME: tumour-associated macrophages (TAMs). TAMs often adopt a pro-tumour (M2) phenotype that promotes tumour growth, angiogenesis, and metastasis. Extracts from *Agrimonia pilosa* have been shown to inhibit the recruitment and pro-tumour functions of TAMs, thereby helping to restore an anti-tumour immune environment [142,143]. While agrimonin and agimonolide-6-O-glucosidehave been observed to modify gut microbiota to generate an antitumor immune response [143], agrimol has also been shown to inhibit mitochondria function in HCT116 cells. This occurs by blocking the peroxisome proliferator-activated receptor-γ coactivator 1α (PGC-1α)/nuclear respiratory factor 1 (NRF1)/mitochondrial transcription factor A (TFAM) pathway subsequently triggering apoptosis both in vitro and in vivo in subcutaneously implanted BALB/c nude mice [144]. Other studies indicate that flavonoids from *A. pilosa* such as quercetin and quercitrin, inhibit the migration and invasion of the RKO colon cancer cell line by modulating the p38, c-Jun N-terminal kinase (JNK), and extracellular signal-regulated kinase (ERK) pathways [145]. The inhibition of angiogenesis and the induction of apoptosis in the CT26 cell line may result from the synergistic action of various *A. pilosa* compounds, including phenolic acids, flavonoids, lignans, and stilbenes [146].

*Patrinia villosa*: The immunomodulatory effects of this plant are mediated through its influence on T-cell populations and cytokine profiles. Studies have shown that treatment with *P.villosa* extracts can modulate the levels of key cytokines such as Interferon-gamma (IFN-γ) and Interleukin-2 (IL-2) and shift the balance of T-cell populations towards a more effective anti-tumour response [147]. *P. villosa* can modulate gut microbiota through its flavonoids; an increased intake of these compounds is known to reduce the risk of colorectal inflammation and cancer [148]. These flavonoids—including quercetin, luteolin, apigenin, kaempferol—are metabolised by *Bacillus Glycinifermentas*, *Flavonifractor plautii*, *Eubacterium eligens*, and *Bacteroides eggerthii* into 2,4,6-trihydroxybenzoic acid and 3,4-dihydroxybenzoic acid, both of which exhibit inhibitory effects on CRC cells [148,149]. Another compound from *P. villosa* generated during boiling is 8,9-didehydro-7-dihydroxydolichodial, which has been shown to reduce the viability of HCT116 cells [150]. Other studies suggest that the anti-CRC activity of *P. villosa* is mediated by the modulation of the PI3K/Akt pathway [151].

Synergy with immunotherapy (PD-1/PD-L1) and microbiome-directed targeted therapies. The TME- and microbiota-modulating agents reviewed in Section 3.4 act on the very molecular axes that determine response to checkpoint inhibitors in mCRC. By depleting *Fusobacterium nucleatum*, restoring SCFA-producing taxa, downregulating TLR4/MYD88-driven autophagy, blunting succinic acid-mediated cGAS–STING suppression, and reducing the TIGIT/CD155 and TIM-3/Galectin-9 immune checkpoint axes, these phytochemicals convert the “cold” immune contexture of microsatellite-stable mCRC into a state more permissive to PD-1 (pembrolizumab, nivolumab) and PD-L1 (atezolizumab, durvalumab) blockade, and to PD-1 + CTLA-4 combinations (nivolumab + ipilimumab). They additionally enhance bevacizumab–atezolizumab and FOLFOXIRI–atezolizumab regimens (AtezoTRIBE) by attenuating VEGF-driven immunosuppression in the hepatic metastatic niche. Thus, every microbiome- or TME-modulating phytochemical proposed here is mechanistically anchored to a specific class of molecular targeted therapy—PD-1/PD-L1 inhibitors, anti-CTLA-4 agents, or anti-VEGF antibodies—rather than acting in isolation [9,10,11,12,13,14,62,63,64,131,132,133,134,142,143].

### 3.5. Reversal of Resistance Pathways and Cancer Stem Cell Suppression: STAT3, CD44/CD133, and Synergy with Molecular Targeted Therapies

Drug resistance to molecularly targeted therapy in mCRC arises through partly overlapping mechanisms that converge on a small number of nodes amenable to phytochemical intervention. Primary resistance to anti-EGFR antibodies is driven by activating mutations in *KRAS*, *NRAS*, *BRAF*, and *PIK3CA* and by HER2/*MET* amplification, while secondary resistance involves the emergence of subclones bearing EGFR ectodomain mutations (e.g., S492R), reactivation of the RAS–RAF–MEK–ERK and PI3K/Akt cascades, EMT, and the autocrine TGF-β/heregulin loop. Resistance to bevacizumab and to anti-angiogenic multikinase inhibitors (regorafenib, fruquintinib) operates through compensatory induction of FGF, PlGF, ANG-2, and HIF-1α-driven hypoxic signalling, with metabolic reprogramming and pericyte recruitment. A unifying feature of both axes is the upregulation of the IL-6/JAK2/STAT3 cascade and ATP-binding cassette efflux pumps (P-glycoprotein/ABCB1, ABCG2/BCRP, MRP1/ABCC1) that extrude cytotoxic and targeted agents. *F. nucleatum* further sustains chemoresistance via TLR4/MYD88-mediated autophagy and succinic acid-dependent suppression of cGAS–STING-driven anti-tumour immunity, thereby linking the microbial, immune, and pharmacological dimensions of resistance [10,11,12,24,25,62,63,64].

STAT3 activation is one of the most consistent molecular fingerprints of cetuximab- and bevacizumab-resistant mCRC. Persistent IL-6/gp130/JAK2 signalling phosphorylates STAT3 at Tyr705, driving the transcription of survival genes (Bcl-xL, Mcl-1, Survivin), of EMT regulators (Twist1, Snail, and ZEB1), of VEGF, and of the stemness factors NANOG, SOX2, and OCT4. Several phytochemicals reviewed above act as direct or upstream STAT3 inhibitors and have demonstrated synergy with targeted agents in preclinical mCRC models: curcumin and its analogue EF24 block JAK2/STAT3 phosphorylation and resensitise *KRAS*-mutant cells to cetuximab; resveratrol abrogates IL-6-induced STAT3 nuclear translocation and potentiates bevacizumab in HCT116 xenografts; EGCG suppresses STAT3-driven Bcl-2 transcription and synergises with regorafenib; baicalein and wogonin from *Scutellaria baicalensis* reduce STAT3 DNA-binding activity and overcome 5-FU resistance; quercetin disrupts the STAT3–HSP90 chaperone interaction and downregulates P-glycoprotein; cucurbitacin B is a covalent JAK2 inhibitor that abrogates STAT3 hyperactivation in *BRAF* V600E lines. The shared endpoint is a coordinated decrease in anti-apoptotic and pro-angiogenic STAT3 targets, providing the mechanistic basis for combining these compounds with cetuximab, panitumumab, bevacizumab, and aflibercept rather than with chemotherapy alone [8,61,82,83,84,85,86,152,153].

Suppression of colorectal cancer stem cells (CSCs) is a second therapeutic axis that targeted therapies do not address effectively. The CSC compartment, identified by combinations of CD44, CD133, LGR5, EpCAM, and ALDH1, sustains tumour-initiating capacity, asymmetric division, chemo- and radio-resistance, and post-treatment relapse [25]. Phytochemicals modulate this compartment through several converging mechanisms. Curcumin and its hexahydro derivatives downregulate CD44 and CD133 expression, inhibit Wnt/β-catenin and Notch signalling, and reduce sphere-forming capacity in HT-29 and HCT116 colonospheres; the curcumin–FOLFOX combination further depletes the CD44+/CD133+ pool in patient-derived xenografts. EGCG suppresses CD133+ subpopulations by inhibiting Wnt/β-catenin and STAT3 and resensitises cells to oxaliplatin. Resveratrol decreases CD44 and ALDH1 activity, induces miR-34a, and represses the SIRT1–c-MYC axis, thereby compromising CSC self-renewal. Sulforaphane from Brassicaceae targets CD44+/CD133+ cells through NF-κB and Hedgehog inhibition. Quercetin and silibinin downregulate CD133 and the ABCG2 efflux pump, restoring sensitivity to irinotecan and 5-FU. Polysaccharides from *Dendrobium candidum* and *Astragalus* modulate the CSC niche via TLR4/NF-κB and STAT3 signalling and amplify the activity of cetuximab in patient-derived organoids. Together, these data identify CD44/CD133-defined CSCs as a tractable, mCRC-relevant target that complements rather than duplicates anti-EGFR and anti-VEGF therapy [25,61,65,82,83,84,85,86,141].

From a translational standpoint, synergism between natural products and molecular targeted therapies in mCRC is therefore best framed not as parallel cytotoxicity but as multi-node convergence on the molecular drivers of metastasis and resistance. (i) On the EGFR/RAS–RAF–MEK–ERK and VEGF axes: grape seed proanthocyanidins, EGCG, and luteolin reduce HIF-1α/VEGF and MMP-2/-9 expression and amplify the anti-angiogenic effects of bevacizumab and aflibercept; curcumin, resveratrol, and silibinin attenuate compensatory EGFR re-phosphorylation and resensitise *KRAS*-mutant or *BRAF*-mutant cells to cetuximab and to the encorafenib + cetuximab regimen used in BEACON CRC. (ii) On the apoptotic/STAT3 axis: curcumin, quercetin, and baicalein restore the Bax/Bcl-2 ratio and caspase-3/9 activation under cetuximab or regorafenib pressure. (iii) On efflux and pharmacokinetic resistance: silymarin, piperine, and quercetin inhibit P-glycoprotein- and CYP3A4-mediated extrusion or metabolism of irinotecan and tyrosine kinase inhibitors, partially restoring intratumoral exposure. (iv) On the immune–microbiota axis: *F. nucleatum*-targeted formulations (berberine, allicin, sodium butyrate-producing prebiotics, polysaccharides from Dendrobium and *Astragalus*) reverse autophagy- and succinic acid-mediated chemo- and immune-resistance and warrant formal evaluation in combination with PD-1/PD-L1 inhibitors in MSS mCRC. Importantly, every phytochemical-targeted-therapy pairing proposed here is anchored in a defined molecular target of the targeted agent (EGFR, VEGF/VEGFR, BRAF, HER2, PD-1/PD-L1, or multikinase) and is intended to extend, not replace, the standard-of-care backbone described in Section 3 [27,28,29,30,62,63,64,66,67,68,69,82,83,84,85,86,152,153]. Notably, only a subset of these resistance-reversal effects has been confirmed in vivo (for example, the curcumin–FOLFOX combination depleting CD44+/CD133+ cells in patient-derived xenografts, and resveratrol potentiating bevacizumab in HCT116 xenografts) whereas the majority rest on cell culture data alone. To make this distinction explicit, the level of evidence for the principal resistance- and CSC-related findings is summarised in Table 3, and the main cancer stem cell-targeting phytochemicals in Table 4.

## 4. Discussion

**Figure 4:**
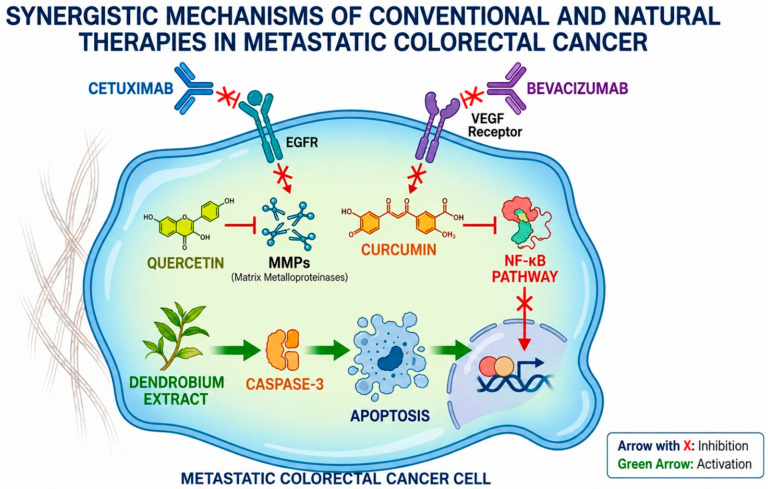
Synergistic Mechanisms of Conventional and Natural Therapies in Metastatic Colorectal Cancer. The figure illustrates the convergent pathways targeted by conventional targeted therapies (cetuximab and bevacizumab) and selected phytochemicals (quercetin, curcumin, and *Dendrobium* extract). Cetuximab blocks the EGFR, while bevacizumab neutralises circulating VEGF-A, indirectly suppressing VEGFR-1/2 activation on endothelial cells. Intracellularly, quercetin inhibits MMP-2/9 and reduces NF-κB-driven transcription; curcumin suppresses NF-κB, PI3K/Akt, and MAPK signalling; and *Dendrobium* polysaccharides shift the Bax/Bcl-2 ratio, activating caspases 3 and 9 along the intrinsic apoptotic pathway. Convergence on the angiogenesis, proliferation, apoptosis, and tumour microenvironment axes provides the mechanistic rationale for rational combination strategies discussed in Section 4. (The figure was generated with Inkscape, versions 0.92 and 1.0).

The management of mCRC has made significant strides with the advent of molecularly targeted therapies, yet substantial challenges remain. Synthesising the data presented in Section 3 and Section 4 and summarised in Table 1 and Table 5 and Figure 4, three principal observations emerge: (a) the survival benefit of approved targeted agents is narrowly confined to molecularly defined subgroups; (b) phytochemical adjuvants act on overlapping but distinct nodes within the same oncogenic networks; and (c) the gut microbiota—most prominently *Fusobacterium nucleatum*—operates as a modifiable determinant of treatment response. This review highlights a critical divergence in the field: while targeted agents like cetuximab offer clear survival benefits in biomarker-selected populations (e.g., *RAS* WT, left-sided tumours), these benefits are often incremental, and issues of toxicity, cost, and inevitable drug resistance persist [154,155]. The FIRE-3 trial exemplifies this paradigm, where a 7-month median OS advantage for cetuximab over bevacizumab in the per-protocol population was not accompanied by a corresponding PFS benefit, suggesting a complex interplay of tumour biology and subsequent therapies [23]. This “PFS-OS paradox” underscores the need for strategies that not only halt progression but also induce deep, durable responses and favourably alter the long-term disease course.

It is in this context that natural products present a compelling, scientifically grounded opportunity. Our review of phytochemicals—quercetin, curcumin, and proanthocyanidins from grape seed extract—reveals a striking mechanistic complementarity to conventional targeted agents (Table 5, Figure 4). While bevacizumab targets the VEGF ligand, compounds like GSE and *Coix lacryma-jobi* inhibit angiogenesis through distinct but related mechanisms (e.g., suppressing the upstream regulator HIF-1α) [67,68,69,74,156]. This suggests the potential for a more comprehensive vertical blockade of the angiogenesis pathway when used in combination. Similarly, while anti-EGFR agents block proliferation signals at the cell surface, phytochemicals like Dendrobium polysaccharides and quercetin induce apoptosis through intrinsic mitochondrial pathways (Bax/Bcl-2) and by inhibiting central inflammatory nodes like NF-κB [81,102,157,158]. This multi-pronged attack on cancer cell survival pathways could theoretically overcome the resistance that emerges from single-target pressure.

The role of the gut microbiota represents another crucial frontier. The high prevalence of *F. nucleatum* in CRC tumours (up to 98% in some cohorts) and its direct link to chemoresistance are major clinical challenges [11,12,62,134,135]. The ability of traditional formulations such as Yanghe decoction and individual compounds to modulate the gut microbiota, increase beneficial species, and enhance anti-tumour immunity offers a novel therapeutic axis [65,141]. By mitigating the pro-tumorigenic and chemoresistant effects of pathogenic bacteria, these natural products could re-sensitise tumours to standard therapies and improve overall treatment efficacy.

However, the translation of these promising preclinical findings into clinical practice is fraught with challenges. The primary obstacle for many phytochemicals, as exemplified by curcumin, is poor oral bioavailability [87]. In the absence of effective delivery systems or formulations, systemic concentrations may never reach the therapeutic levels (e.g., 20–35 μM for quercetin) required to exert an anti-cancer effect outside the gastrointestinal tract [103,104]. This necessitates a greater focus on pharmaceutical development, including nano-formulations and other delivery technologies, to unlock the systemic potential of these compounds [104,159,160]. Furthermore, the lack of standardisation and the inherent variability of natural extracts pose significant hurdles for rigorous clinical investigation and regulatory approval.

Future research must move beyond single-agent preclinical studies and focus on well-designed clinical trials investigating rational combinations of phytochemicals and targeted therapies. Such trials should incorporate robust biomarker strategies, including gut microbiome profiling, to identify patient populations most likely to benefit. Investigating the impact of these combinations on quality of life, toxicity profiles, and cost-effectiveness will be paramount for their integration into standard oncology practice.

### 4.1. Bioavailability and Drug Delivery

A major translational hurdle for many phytochemicals is their limited oral bioavailability. To address this, research is actively exploring advanced drug delivery systems. Nano-formulations, such as liposomes, nanoparticles, and phytosomes, have shown significant promise in improving the solubility, stability, and systemic absorption of compounds like curcumin and quercetin [87,90,91,93,94,95,104,159,160]. These technologies can protect phytochemicals from degradation in the gastrointestinal tract and facilitate their transport across cellular membranes, thereby increasing their concentration at the tumour site and enhancing their therapeutic efficacy.

### 4.2. Herb–Drug Interactions

The potential for herb–drug interactions (HDIs) is a critical safety consideration when integrating phytochemicals into conventional oncology regimens. Many phytochemicals, including quercetin, are known to inhibit cytochrome P450 (CYP) enzymes, such as CYP3A4, and drug transporters like P-glycoprotein [152,153]. Since many chemotherapeutic agents are metabolised by these same pathways, co-administration may alter their pharmacokinetics, potentially leading to increased toxicity or reduced efficacy. Therefore, careful monitoring and further research into the clinical significance of these interactions are essential before combination therapies can be widely adopted.

Hepatic metabolism and hepatotoxicity deserve particular attention in mCRC because the liver is simultaneously the dominant metastatic site, the principal organ of biotransformation for fluoropyrimidines, irinotecan, oxaliplatin, regorafenib, encorafenib, and most tyrosine kinase inhibitors, and a frequent target of treatment-induced injury. Oxaliplatin causes sinusoidal obstruction syndrome (SOS, “blue liver”) in up to 50% of patients receiving prolonged FOLFOX, while irinotecan-based regimens are associated with chemotherapy-associated steatohepatitis (CASH, “yellow liver”) in 10–20% of patients; bevacizumab partially mitigates SOS but increases peri-operative bleeding risk, and regorafenib carries a class warning for grade 3–4 hepatotoxicity. Phytochemicals interact with this hepatic compartment in two opposing ways. On one hand, polyphenols (quercetin, resveratrol, EGCG, silibinin, curcumin), grapefruit furanocoumarins, St John’s wort hyperforin, and *Astragalus* saponins modulate CYP3A4, CYP2C9, CYP1A2, UGT1A1, and the P-glycoprotein/BCRP transporters and can either potentiate (irinotecan→SN-38 via UGT1A1 inhibition; regorafenib via CYP3A4 inhibition) or attenuate (5-FU via DPD modulation; cetuximab antibody-dependent cellular cytotoxicity) clinical exposure, with effects that are highly dose- and formulation-dependent. On the other hand, several of the same compounds exert hepatoprotective effects of direct relevance to mCRC patients: silymarin/silibinin from *Silybum marianum* reduces oxaliplatin-induced SOS and CASH in randomised pilot studies; curcumin, EGCG, and resveratrol attenuate hepatic oxidative stress, lipid peroxidation, and TNF-α/IL-6-mediated liver injury through Nrf2 activation and NF-κB inhibition; and glycyrrhizin reduces transaminase elevations during chemotherapy. From a clinical-pharmacology standpoint, any phytochemical considered as an adjuvant in mCRC should therefore be characterised pharmacokinetically alongside the targeted agent it is intended to support, with explicit monitoring of liver enzymes, coagulation, and bilirubin, and with avoidance of concomitant strong CYP3A4-modulating botanicals (St John’s wort, high-dose grapefruit) during cetuximab-, irinotecan-, regorafenib-, or encorafenib-based regimens [152,153]. More broadly, several natural and adjunctive agents display a favourable dual (pleiotropic) profile, selectively enhancing cytotoxicity in tumour cells while protecting normal tissues from treatment-related injury: for example, *Hibiscus sabdariffa* extract reduced cisplatin-induced hepatotoxicity in vivo while simultaneously increasing cisplatin cytotoxicity in cancer cells [31], and incretin-based agents attenuated doxorubicin-induced nephrotoxicity through SIRT1/Nrf2/NF-κB modulation [161]. Such organ-protective antioxidant strategies are directly relevant to mitigating the cumulative toxicity of mCRC regimens and merit systematic evaluation alongside targeted therapy.

### 4.3. Translational Barriers and Limitations

Despite the breadth of the available evidence, several issues remain unsolved and frame the translational barriers discussed below. First, even within the “left-sided *RAS* wild-type” subgroup, in which anti-EGFR therapy is most active, primary or acquired resistance ultimately develops in the majority of patients, and no validated strategy currently reverses it durably. Second, the molecular and cytological hallmarks that distinguish metastasis-competent CRC clones—loss of *APC* and *SMAD4*, *TP53* mutation, MSI/MMR status, *KRAS*/*NRAS*/*BRAF*/*PIK3CA* co-mutations, EMT, expansion of CD44+/CD133+/LGR5+ cancer stem cell compartments, and a *Fusobacterium nucleatum*-enriched microenvironment—have not yet been integrated into a single therapeutic framework that also accommodates phytochemical adjuvants. Third, the available evidence on natural products is fragmented, with limited data on hepatic metabolism, hepatotoxicity, and herb–drug interactions in the very organ that is also the dominant metastatic site of CRC. This review specifically addresses these gaps by mapping mCRC-specific molecular nodes (RAS–RAF–MEK–ERK, PI3K/Akt, IL-6/JAK2/STAT3, Wnt/β-catenin, HIF-1α/VEGF, MMP-2/-9, ABC transporters, and CD44/CD133 stemness) to natural product chemistry and to the synergistic combinations that are mechanistically plausible with cetuximab, panitumumab, bevacizumab, aflibercept, regorafenib, encorafenib, trastuzumab, and PD-1/PD-L1 inhibitors.

Several methodological caveats temper the conclusions drawn from the available preclinical literature on plant-derived adjuvants in mCRC. First, the majority of mechanistic data derive from a restricted panel of established cell lines (HCT116, HT-29, SW480, SW620, Caco-2), whose mutational profiles do not capture the full molecular heterogeneity of clinical mCRC; in particular, *BRAF* V600E and HER2-amplified phenotypes—both increasingly relevant to therapeutic decision-making—remain underrepresented in phytochemical synergy studies [15,19,52]. Second, in vivo confirmation is frequently limited to subcutaneous xenograft models in immunodeficient mice, which inadequately reproduce the hepatic and peritoneal metastatic niches that dominate mCRC clinical biology [38,57]. Third, the concentrations at which synergy with 5-FU, oxaliplatin, or cetuximab is demonstrated in vitro (typically 10–50 μM for quercetin and curcumin) substantially exceed the systemic exposures achievable with current oral formulations, raising legitimate questions about clinical translatability beyond the colonic luminal compartment [87,103,104]. Fourth, heterogeneity in extract standardisation, solvent systems, and reported active fractions across studies precludes formal meta-analytic synthesis and complicates reproducibility. Finally, prospective randomised data evaluating phytochemical adjuvants alongside contemporary first-line regimens (FOLFOX/FOLFIRI ± cetuximab or bevacizumab) remain scarce, and the few completed trials have generally been single-arm or underpowered for survival endpoints. These limitations should be considered when interpreting the mechanistic complementarity highlighted throughout this review. Ranking the reviewed phytochemicals by the current strength of evidence is therefore useful for prioritisation. Curcumin, quercetin, resveratrol, EGCG, and grape seed proanthocyanidins are the best supported, combining reproducible mechanistic data with early phase human pharmacokinetic or chemoprevention studies; sulforaphane, silibinin, and berberine occupy an intermediate tier with consistent in vitro and in vivo activity but minimal clinical data; and most plant- or formulation-specific extracts (Dendrobium and *Astragalus* polysaccharides, *Paris polyphylla* saponins, *Coix lacryma-jobi*, *Mesua ferrea*, *Ranunculus ternatus*, *Momordica balsamina*, *Patrinia villosa*, *Agrimonia pilosa*, and traditional formulations such as Yanghe decoction) remain at the preclinical, hypothesis-generating stage. This hierarchy should guide the selection of candidates for clinical translation.

From a translational and intellectual property perspective, the patent landscape for natural product adjuncts in mCRC remains comparatively underdeveloped. Although numerous patents cover phytochemical compositions, nano-formulations, and standardised extracts of curcumin, quercetin, resveratrol, EGCG, and grape seed proanthocyanidins for broad oncological or anti-inflammatory indications, filings that claim specific, mechanism-defined combinations of these agents with approved targeted antibodies (cetuximab, panitumumab, bevacizumab) or with multikinase inhibitors in colorectal cancer are scarce. This relative gap reflects the early translational maturity of the field but also represents an opportunity: the rationally designed, biomarker-anchored phytochemical-targeted therapy combinations outlined here are potentially patentable and could help incentivise the formal clinical development that the area currently lacks.

### 4.4. Future Perspectives

Translation of the mechanistic findings reviewed here into clinical practice will require a coordinated research agenda along four converging axes. (i) Pharmaceutical development: nano-enabled delivery platforms (liposomes, polymeric nanoparticles, phytosomes, and self-emulsifying drug delivery systems) should be prioritised for compounds with documented preclinical activity but limited oral bioavailability, with particular attention to tumour-targeted formulations capable of achieving therapeutically relevant intratumoural concentrations [87,90,91,93,159,160]. (ii) Biomarker-guided trial design: future early phase studies of phytochemical-targeted agent combinations should incorporate prospective stratification by *RAS*/*BRAF* status, primary tumour sidedness, MSI/MMR status, and baseline gut microbiome composition—including *Fusobacterium nucleatum* tumoural load—to identify the patient subsets most likely to derive benefit [138,139]. (iii) Microbiome-modulatory strategies: rational integration of dietary phytochemicals, prebiotics, and selectively targeted antimicrobials offers a plausible route to attenuate *F. nucleatum*-mediated chemoresistance and immune evasion and warrants formal evaluation in combination with PD-1/PD-L1 inhibitors in MSS mCRC [62,63,64]. (iv) Real-world and digital evidence: integration of electronic health records, registry data, and emerging digital twin and AI-enhanced platforms—already piloted in precision oncology [8,26]—could accelerate hypothesis generation regarding herb–drug interactions and outcome modifiers in routine practice, complementing the necessarily restricted scope of randomised trials. Collectively, these directions reframe phytochemicals from passive nutraceuticals to active, mechanistically defined components of an integrated, personalised therapeutic armamentarium for mCRC.

## 5. Conclusions

The treatment landscape of mCRC is evolving towards a more personalised and integrated model. While molecularly targeted therapies have significantly improved outcomes for biomarker-defined subgroups, they are not a panacea. The evidence reviewed here strongly suggests that plant-derived phytochemicals are not merely “alternative” medicines but are potent biological response modifiers with clear, scientifically validated mechanisms of action that complement conventional drugs. By inhibiting angiogenesis, inducing apoptosis, suppressing metastasis, and modulating the tumour microenvironment and gut microbiota, these compounds have the potential to synergistically enhance the efficacy of targeted therapies, overcome drug resistance, and improve patient outcomes. The integration of evidence-based natural products into the continuum of mCRC care represents a logical, sustainable, and highly promising strategy to address the current limitations of oncology.

## Figures and Tables

**Table 1:** Comparison of Key Phase III Trials for First-Line Targeted Therapies in RAS Wild-Type mCRC.

Trial	N (*RAS* WT)	Treatment Arms	Median OS	HR (OS) [95% CI]	Median PFS	HR (PFS) [95% CI]	ORR
CRYSTAL [39]	~667	Cetuximab + FOLFIRI vs. FOLFIRI	23.5 vs. 20.0 mo	0.84 [0.70–0.99]	9.9 vs. 8.4 mo	0.68 [0.50–0.94]	57.3% vs. 39.7%
FIRE-3 [23]	400	Cetuximab + FOLFIRI vs. Bevacizumab + FOLFIRI	31 vs. 26 mo	0.76 (*p* = 0.012)	10.0 vs. 10.3 mo	1.06 (*p* = 0.55)	77% vs. 65%
CALGB 80405 [42]	1137	Cetuximab + Chemo vs. Bevacizumab + Chemo	30.0 vs. 29.0 mo	0.92 [0.78–1.09]	10.5 vs. 10.6 mo	0.95 [0.82–1.09]	60% vs. 54%

Note: Data represent outcomes in the *RAS* wild-type populations. Chemo in CALGB 80405 was the investigator’s choice of FOLFOX or FOLFIRI. OS in FIRE-3 is for the overall *RAS* WT population; the benefit was concentrated in left-sided tumours.

**Table 2:** Approximate frequencies of recurrent molecular alterations in metastatic colorectal cancer and their principal therapeutic relevance.

Gene/Alteration	Approx. Frequency in mCRC	Principal Clinical/Therapeutic Relevance
*APC* (loss-of-function)	~75–80%	Initiates Wnt/β-catenin activation; enriched at the invasive front
*TP53* mutation	50–60%	Adenoma-to-carcinoma transition; associated with hepatic metastatic spread
*KRAS* (incl. G12C ~3–4%)	~40–45%	Predicts anti-EGFR resistance; G12C druggable with sotorasib/adagrasib plus an anti-EGFR antibody
*NRAS*	~5%	Predicts anti-EGFR resistance (part of extended *RAS* testing)
*BRAF* V600E	8–12%	Right-sided, MSI-prone, poor prognosis; encorafenib + cetuximab (BEACON CRC)
*PIK3CA*	15–18%	Engages PI3K/Akt/mTOR survival signalling; frequently co-occurs with *RAS*
*SMAD4* (inactivation)	10–20%	TGF-β pathway dysregulation; predicts distant metastasis and poor anti-EGFR response
HER2 (*ERBB2*) amplification	3–5%	Actionable with trastuzumab + tucatinib (MOUNTAINEER) or trastuzumab–deruxtecan
dMMR/MSI-H	4–5%	Hypermutated, high TMB; responsive to immune checkpoint inhibitors (KEYNOTE-177)
*NTRK*/*ALK*/*ROS1* fusions	<1%	Rare but actionable with larotrectinib/entrectinib
*POLE*/*POLD1* (ultramutated)	<1%	Very high TMB; candidate for immune checkpoint blockade

Frequencies are approximate and vary across cohorts and detection platforms. TMB, tumour mutational burden; dMMR/MSI-H, deficient mismatch repair/microsatellite instability-high.

**Table 3:** Level of evidence for representative resistance-reversal and cancer stem cell (CSC) findings discussed in Section 3.5.

Finding (Representative)	Principal Mechanism	Highest Evidence Level
Curcumin + FOLFOX depletes the CD44+/CD133+ CSC pool	Wnt/β-catenin and Notch inhibition; CD44/CD133 downregulation	Animal (patient-derived xenograft)
Resveratrol potentiates bevacizumab	IL-6/STAT3 inhibition; anti-angiogenic	Animal (HCT116 xenograft)
Quercetin/sulforaphane enhance oxaliplatin	GSH depletion; Bax/Bcl-2 shift	Animal (HCT116 xenograft)
Curcumin/EGCG resensitise *KRAS*/*BRAF*-mutant cells to cetuximab	NF-κB and PI3K/Akt suppression	Cell culture
Quercetin/silibinin reduce P-glycoprotein/ABCG2 efflux	Efflux-pump inhibition	Cell culture
Dendrobium/*Astragalus* polysaccharides amplify cetuximab	TLR4/NF-κB and STAT3 modulation	Cell culture (organoids)
Curcumin colonic activity/chemoprevention	Local NF-κB and gut–microbiota modulation	Clinical (early phase)

**Table 4:** Cancer stem cell (CSC)-targeting phytochemicals in colorectal cancer.

Phytochemical	CSC Marker/Pathway Targeted	Reported Effect	Evidence Level
Curcumin	CD44, CD133; Wnt/β-catenin, Notch	Reduced sphere formation; CSC pool depletion	Cell and animal (PDX)
EGCG	CD133; Wnt/β-catenin, STAT3	Reduced CD133+ fraction; oxaliplatin resensitisation	Cell culture
Resveratrol	CD44, ALDH1; SIRT1–c-MYC, miR-34a	Impaired self-renewal	Cell culture
Sulforaphane	CD44/CD133; NF-κB, Hedgehog	Reduced CSC viability	Cell culture
Quercetin/silibinin	CD133; ABCG2	Restored irinotecan/5-FU sensitivity	Cell culture
Dendrobium/*Astragalus* polysaccharides	CSC niche; TLR4/NF-κB, STAT3	Amplified cetuximab activity in organoids	Cell culture (organoids)

**Table 5:** Summary of mechanisms for selected plant-derived compounds in mCRC.

Compound/Plant	Primary Mechanism	Key Molecular Targets	Potential Synergy
*Vitis vinifera* (GSE)	Anti-Angiogenesis	VEGF, HIF-1α, MMPs	With Bevacizumab and Chemotherapy
*Coix lacryma-jobi*	Anti-Angiogenesis	Angiogenic factor secretion	With Bevacizumab and Regorafenib
*Dendrobium candidum*	Apoptosis Induction	Bax/Bcl-2 ratio, Caspase-3/9	With Chemotherapy (e.g., 5-FU)
Curcumin	Apoptosis, Anti-Proliferation	NF-κB, PI3K/Akt, MAPK	With Chemotherapy and Targeted Agents
Quercetin	Apoptosis, Anti-Metastasis	NF-κB, MMP-2/9, E-cadherin	With 5-FU and Oxaliplatin
*Mesua ferrea*	Anti-Metastasis	WNT, HIF-1α, EGFR	With Cetuximab and Chemotherapy
Yanghe Decoction	Microbiota/Immune Modulation	*Bacillus* spp., NK cells, IL-21	With Immunotherapy and Chemotherapy
*Agrimonia pilosa*	TME Modulation	Tumour-Associated Macrophages (TAMs)	With Immunotherapy (e.g., PD-1 inhibitors)

## Data Availability

No new data were created or analysed in this study. Data sharing is not applicable to this article.

## Abbreviations

The following abbreviations are used in this manuscript:

5-FU	5-fluorouracil
AMPK	AMP-activated protein kinase
CASH	chemotherapy-associated steatohepatitis
CBD	cannabidiol
CLJ	*Coix lacryma-jobi*
CLSE	adlay seed extract
CRC	colorectal cancer
CSC	Cancer stem cell
ctDNA	circulating tumour DNA
mCRC	metastatic CRC
dMMR/MSI-H	deficient mismatch repair/microsatellite instability-high
EGCG	epigallocatechin-3-gallate
EGFR	epidermal growth factor receptor
EMT	epithelial–mesenchymal transition
ERK	extracellular signal-regulated kinase
FGFR	fibroblast growth factor receptor
FMT	faecal microbiota transplantation
FOLFIRI	fluorouracil, leucovorin, irinotecan
FOLFOX	fluorouracil, leucovorin, oxaliplatin
GSE	grape seed extract
HDI	herb–drug interaction
HIF-1α	hypoxia-inducible factor-1α
IFN-γ	interferon γ
IL	interleukin
JNK	c-Jun N-terminal kinase
MMP	metalloproteinase
MSI	microsatellite instability
NF-kB	nuclear factor-kB
NK	natural killer
NRF1	nuclear respiratory factor 1
ORR	objective response rate
OS	overall survival
PD-1	programmed cell death protein-1
PDGFR	platelet-derived growth factor receptor
PFS	progression-free survival
PGC-1α	peroxisome proliferator-activated receptor γ coactivator 1α
PlGF	placental growth factor
RCTs	randomised controlled trials
ROS	reactive oxygen species
SOS	sinusoidal obstruction syndrome
TFAM	mitochondrial transcription factor A
THC	tetrahydrocannabinol
TLR3	Toll-like receptor 3
TMB	tumour mutational burden
TME	tumour microenvironment
VEGF	vascular endothelial growth factor
WT	wild-type
xIAP	X-linked inhibitor of apoptosis protein
